# Free Preconceptual Screening Examination Service in Rural Areas of Hubei Province, China in 2012

**DOI:** 10.1371/journal.pone.0111918

**Published:** 2014-11-06

**Authors:** Cui-ling Li, Kai Zhao, Hui Li, Omar Ibrahim Farah, Jiao-jiao Wang, Rong-ze Sun, Hui-ping Zhang

**Affiliations:** 1 Family Planning Research Institute, Tongji Medical College, Huazhong University of Science and Technology, Wuhan, Hubei, China; 2 Department of Science and Technology Service, Hubei Provincial Population and Family Planning Commission, Wuhan, Hubei, China; 3 Renmin Hospital of Wuhan University, Wuhan, Hubei, China; Vanderbilt University, United States of America

## Abstract

**Objective:**

This work aims to collect and summarize the outcomes on free preconceptual screening examination in rural areas of Hubei Province in 2012. Moreover, this review promotes further understanding of the status of this activity to provide the Family Planning Commission valid scientific data upon which to construct effective policies.

**Methods:**

Couples, who complied with the family planning policy and were the residents in agricultural areas or lived in a local rural area for more than six months, were encouraged to participate in the free preconceptual screening examination service provided by the Hubei Provincial Population and Family Planning Commission. This service included 19 screening tests. All the data, including forms, manuals, and test results, were collected from 1 January 2012 to 31 December 2012 in rural areas in Hubei Province.

**Results:**

A total of 497,860 individuals participated in the free preconceptual screening examination service, with a coverage rate of 97.1%. 4.0% and 4.8% of the participants exhibited with abnormal blood levels of ALT and creatinine, respectively; 0.36% of the participants tested positive for syphilis; 0.44% and 3.6% of the female participants tested positive for *Neisseria gonorrhoeae* and *Chlamydia trachomatis*, respectively; and 0.84% and 1.8% of the female participants tested positive for cytomegalovirus (IgM) and *Toxoplasma gondii* (IgM), respectively. After risk assessment, 59,935 participants might have high-risk of adverse pregnancy outcomes. In 2012, the prevalence of birth defects among the parturient who participated in the preconceptual screening examination service was 0.04%, while the prevalence was 0.08% among those who did not participate in the service.

**Conclusion:**

Preconceptual screening examination service may help to address the risk factors that can lead to adverse pregnancy outcome. More studies on the relationship between preconceptual screening examination service and prevalence of birth defect or other adverse pregnancy outcomes should be conducted.

## Introduction

A birth defect is an abnormality that is present at birth, such as a missing limb, malformed heart, or Down's syndrome. Birth defects occur in approximately 6% of births worldwide each year [1]. In China, the birth defect incidence is 4% to 6% [2], and the prevalence in 2008 was 1.4% in Hubei Province [3]. In China, there are some free basic public health service for the people that are provided by the government, such as tuberculosis treatment, HIV screening and treatment, vaccinations, infectious disease monitoring, maternal and child health care, seniors health care, and health education. Women can access to the maternal and child health care service in local medical health care organizations for free [4]. However, maternal and child health care are carried out in the antepartum and postpartum period, which is consistent with the concept of primary prevention. The concept of preconception health care has been articulated for over a decade but are yet to become part of free medical service in China. Preconception health care is a preventive strategy that helps men and women prepare for pregnancy by improving their health prior to conception, including health practices related to safeguarding fertility, preparing for pregnancy, and identifying and addressing risk factors [5]–[8]. Thus, preconception health care is important to improve pregnancy outcomes and birth quality.

Considering the importance of preconception health care, the National Population and Family Planning Commission officially launched the national project for the free preconceptual screening examination in China on 22 April 2010. The National Population and Family Planning Commission selected 18 provinces as the first batch of pilot provinces to carry out this national project. While there are many screening tests that are related with the pregnancy and preconception health, the National Population and Family Planning Commission set 19 items of screening tests that are provided in the preconceptual screening examination project due to they are the basic examinations and known associations with adverse pregnancy outcomes. Hubei Province is one of the 18 pilot provinces. Hubei Province is the 14th largest province with the ninth largest population in China (57,237,727 individuals, 2010) [9]. Since 22 April 2010, the Hubei Provincial Population and Family Planning Commission (HPPFPC) had employed the free preconceptual screening examination project to promote the comprehensive development of people, balanced development of the population, and healthy development of the family. Depending upon locality in rural areas, this project aimed to provide free preconceptual screening examination service for 256,274 pairs of rural couples (512,548 individuals) in Hubei Province in 2012.

The main objectives of the project included the following: to improve the quality of births, reduce the prevalence of birth defects, improve the knowledge of preconception health care among couples, enhance the consciousness and initiative of couples to participate in this project, and enhance the competence of primary service agencies to conduct the primary prevention of birth defects. Improving preconception health care can improve pregnancy outcomes by improving the overall health of men and women [10], [11]. Screening by a knowledgeable professional can identify unrecognized risks to the mother and child [12]. Preconception counseling can increase men and women's knowledge and, more importantly, the likelihood that they will make any behavioral changes needed before and during pregnancy to reduce the risk of adverse pregnancy outcomes.

Many studies document the effectiveness of interventions targeted at increasing awareness of preconception folic acid supplementation. However, limited evidence links comprehensive preconception health care promotion to improve pregnancy outcomes [13]–[16]. In this study, data were collected and summarized to understand the status of the preconceptual screening examination work in Hubei Province. The pregnancy outcomes among the pregnant and parturient who participated in and who did not participate in the preconceptual screening examination service in 2012 were compared and analyzed. Greater understanding of these concepts will lead to better informed strategies by researchers, policy makers, and clinicians to assist men and women to optimize preconception health and reduce adverse pregnancy outcomes.

## Materials and Methods

### Target service population

The target population who were eligible to participate in the free preconceptual screening examination service met the following conditions:

- 1). Couples who complied with the family planning policy and prepared for pregnancy in 2012.
- 2). A couple, at least one of whom was a resident in an agricultural area or was defined as a rural resident.
- 3). A couple, at least one of whom was a local residence. Alternatively, none of them was a local resident, but both lived in the locale for more than six months.

### Data source

A list of all eligible individuals was obtained from the Ministry of Civil Affairs and local family planning institutions. All the couples were confirmed to be married in accordance with the data of Ministry of Civil Affairs. Couples who prepared for pregnancy registered in the local family planning institutions and obtained a birth certificate before pregnancy. From 1 January 2012 to 31 December 2012, a total of 256,274 pairs of couples in rural areas of 46 pilot counties in Hubei Province met the conditions that the free preconceptual screening examination service required. All the data, including forms, manuals, test results and pregnancy follow-ups record, were collected from 1 January 2012 to 31 December 2012 in rural areas in Hubei Province.

Additional data specific to the pregnancy outcomes of 177416 pregnant and parturient who did not participate in the preconceptual screening examination service were collected from 1 January 2012 to 31 December 2012, and were obtained from the department of Obstetrics and Gynecology in the local hospitals in other 21 counties in Hubei Province.

### Service content

The service provided 19 items of preconception health care screening tests, including health education on childbearing knowledge, history taking, physical examination, reproductive examination, clinical laboratory tests, risk assessment, and counseling and guidance for both male and female participants. History taking included birth history, history of diseases, family history, medication, lifestyle, diet and nutrition, and environmental risk factors. Physical examination included height, weight, blood pressure, heart rate, thyroid palpation, heart lung auscultation, palpation of liver and spleen, and limb and spinal column inspection. Reproductive examination included inspection and palpation of the external genitalia. Clinical laboratory tests included urinalysis, blood type (ABO type and Rh type), glutamin-pyruvic transaminase (ALT), hepatitis B serologic markers, creatinine, and syphilis screening.

Moreover, some were tests only for female participants, including gynecological ultrasound examination, vaginal leucorrhea exam, *Neisseria gonorrhoeae* and *Chlamydia trachomatis*, complete blood count (CBC), serum glucose, thyroid-stimulating hormone (TSH), rubella virus (IgG), cytomegalovirus (IgM and IgG), *Toxoplasma gondii* (IgM and IgG), early pregnancy follow-up, and pregnancy outcome follow-up. Early pregnancy follow-up was carried out within 12 weeks of pregnancy, and pregnancy outcome follow-up was within 6 weeks after childbirth or within 2 weeks after other terminated pregnancy. The main objective of early pregnancy follow-up was to inform precautions during pregnancy and prenatal care, and to give necessary health guidance and counseling. Pregnancy outcomes, such as normal newborn, preterm birth, low birth weight, androgyneity, birth defect, spontaneous abortion, elective abortion, abortion by labor induction, ectopic gestation, and fetal death/stillbirth were recorded in the Pregnancy Outcome Record during the pregnancy outcome follow-up. Sex, birth weight, gestational age and physical examination of the newborn were also recorded.

### Service agencies

Preconceptual screening examination service were provided by the county-level family planning institutions that obtained the Practice License of the Family Planning Technical Institutions and Medical Institutions Permit.

Engaging in health education work were the health care personnel who received professional training. Engaging in history taking, physical and reproductive examination, ultrasound, clinical laboratory tests, risk assessment, and counseling and guidance were the licensed physicians or physician's assistants. The congenital anomalies and adverse pregnancy outcomes were diagnosed and recorded by the doctors working the department of Obstetrics and Gynecology.

### Financial support

The elementary cost was RMB 240 ($ 39.45), including female RMB 190 ($31.24) and male RMB 50 ($8.21), per child time for the preconceptual screening examination service for every couple. Approximately 50% of the cost was supported by national finance, 30% was provided by provincial public finance, and 20% was supported by county-level public finance.

### Information management

1) Information collection

Data on the free preconceptual screening examination service were collected from 1 January 2012 to 31 December 2012 in rural areas of 46 pilot counties in Hubei Province. The data on eligible couples within the jurisdiction were collected and reported by the family planning administrators in the village (community) to the township (town, street) family planning office. Data were subsequently reported to the family planning institutions at the county level. Finally, all data were collected and reported to the HPPFPC.

A Family Archive was established for each eligible couple. All documents in the Family Archive included the Information Consent Form, service record manual, tests results, risk assessment guidance document, records of early pregnancy follow-up, and pregnancy outcome follow-up. All data were encoded into a network system called “National Project of Free Preconceptual Screening Examination Service Information Management System”.

2) Data quality control

At the first week of each month, the personnel working in local family planning institutions used the “National Project of Free Preconceptual Screening Examination Service Information Management System” to generate the reports and statistics, which handed over to the HPPFPC. The personnel working in HPPFPC organized spot checks once every three months to ensure the accuracy, authenticity and reliability of the data.

### Ethical review

The study protocol was approved by the institutional review boards of the Tongji Medical College, Huazhong University of Science and Technology. All the participants signed the informed written consent after the personnel explained the study protocol.

## Results

### Number of women participating in each pregnancy-related service

From 1 January 2012 to 31 December 2012, a total of 256,274 couples (512,548 individuals) were eligible to participate in the free preconceptual screening examination service in rural areas of 49 pilot counties in Hubei, whereas 497,860 individuals participated in the service, with a coverage rate of 97.1% (497,860/512,548). According to the results of all tests, participants were divided into two groups: general population and high-risk population. Participants with one or more abnormal history, physical examination and test results that might result in a birth defect or other adverse pregnancy outcomes were grouped to the high-risk population. After the risk assessment, 59,935 participants (12.0%) were grouped to the high-risk population, among whom 40.5% were male and 59.5% were female. A total of 86,970 pregnant women participated in the early pregnancy follow-up, and 55,136 pregnant and parturient participated in the pregnancy outcome follow-up (Table 1).

**Table 1:** Individuals Who Participated in the Preconception Health Care Check-up.

Individuals	Male	Percent (%)	Female	Percent (%)	In Total (per child time)
Participant	242578	48.7	255282	51.3	497860
Health Education on Childbearing knowledge	—	—	—	—	596858
High-risk Population	24273	40.5	35662	59.5	59935
Counseling and Guidance	—	—	—	—	494587
Early Pregnancy Follow-up	—	—	86970	—	86970
Pregnancy Outcome Follow-up	—	—	55136	—	55136

### Results of tests for both male and female participants

A total of 496,331, 497,359, and 490,427 participants took the history taking, physical examination, and reproductive examination, respectively, among whom 19.6%, 13.0%, and 6.6% showed abnormalities. Moreover, 4.0% of the participants exhibited an abnormal blood level of ALT, and 4.8% exhibited an abnormal blood level of creatinine. In addition, 0.36% of the participants tested positive for syphilis (Table 2).

**Table 2:** Results of Tests for both Male and Female individuals.

Individuals	Normal	Percent (%)	Abnormal	Percent (%)	In Total (per child time)
History Taking	399197	80.4	97134	19.6	496331
Physical Examination	432461	87.0	64898	13.1	497359
Reproductive Examination	458160	93.4	32267	6.6	490427
Urinalysis	463898	95.6	21234	4.4	485132
Glutamin-Pyruvic Transaminase	471441	96.0	19711	4.0	491152
Hepatitis B Serologic Markers*	452449	91.9	39713	8.1	492162
Creatinine	465701	95.2	23598	4.8	489299
Syphilis Test*	465303	99.6	1681	0.36	466984
	**Ordinary Blood Type**	**Percent (%)**	**Special Blood Type**	**Percent (%)**	**In Total (per child time)**
Blood Type	474360	98.3	8053	1.7	482413

*Hepatitis B Serologic Markers: includes HBs-Ag, HBs-Ab, HBe-Ag, HBe-Ab and HBc-Ab; **Syphilis Test:** *Treponema pallidum* Hemagglutination Assay.

### Results of the tests only for female participants

A total of 248,385 and 247,616 female participants were tested for *Neisseria gonorrhoeae* and *Chlamydia trachomatis*, respectively, among whom 0.44% and 3.6% tested positive. Moreover, 7.7% of the female participants had abnormal serum glucose, and 6.6% exhibited abnormal blood levels of TSH. In addition, 0.84% and 1.8% of female participants tested positive for cytomegalovirus (IgM) and *Toxoplasma gondii* (IgM) tests, respectively (Table 3).

**Table 3:** Results of Tests Only for Female Individuals.

Individuals	Normal	Percent(%)	Abnormal	Percent(%)	In Total (per child time)
Gynecological Ultrasound Examination	248687	96.6	8840	3.4	257527
Vaginal Leucorrhea Exam	234095	93.1	17395	6.9	251490
Serum Glucose	248737	92.3	20877	7.7	269614
Complete Blood Count	220258	79.6	56328	20.4	276586
Thyroid-stimulating Hormone	247348	93.4	17397	6.6	264745

	Negative	Percent(%)	Positive	Percent(%)	In Total
*Neisseria Gonorrheae*	247304	99.6	1081	0.44	248385
*Chlamydia Trachoatis*	238677	96.4	8939	3.6	247616
Rubella Virus IgG	124033	47.7	136105	52.3	260138
Cytomegalovirus IgM	254650	99.2	2150	0.84	256800
Cytomegalovirus IgG	146142	57.1	109804	42.9	255946
*Toxoplasma Gondii* IgM	251122	98.2	4693	1.8	255815
*Toxoplasma Gondii* IgG	245389	95.9	10391	4.1	255780

### Pregnancy outcomes among the pregnant and parturient who participated in and who did not participate in preconceptual screening examination

In 2012, a total of 60,398 pregnant and parturient (including some pregnant and parturient who participated in the service in 2011 and delivered in 2012) from 46 pilot counties, who participated in the preconceptual screening examination service delivered or terminated the pregnancy; while a total of 177,416 pregnant and parturient from other 21 counties, who did not participate in the service, delivered or terminated the pregnancy. The general information of pregnancy outcomes follow-up individuals who participated or did not participate in the service were shown in Table 4, including age, minority, smoking status and education level. There was no significant difference between the pregnant and parturient who participated or did not participate in the service. The prevalence of birth defects was 0.04% among 58,928 follow-up newborns, while the prevalence was 0.08% among those whose parturient did not participate in the service (Table 5). The prevalence of spontaneous abortion, ectopic gestation, and fetal death/stillbirth among 60,398 pregnant and parturient were 1.3%, 0.07%, and 0.13%, respectively (Table 5). The prevalence of birth defects among the parturient who participated in the service was lower than that among the parturient who did not (χ^2^ = 10.7, *P* = 0.001). Also, the prevalence of other adverse pregnancy outcomes was lower among the pregnant and parturient who participated in the service (χ^2^ = 1820.2, *P*<0.001) (Table 5).

**Table 4:** General Information Distribution among Pregnancy Outcomes Follow-up Individuals.

	Individuals who participated in the service		Individuals who did not participate in the service		
	(n = 58928)		(n = 166819)		
	Number	Percent (%)	Number	Percent(%)	*P* value
**Age**					
20–24	7778	13.2	22353	13.4	*P*>0.05
25–29	29641	50.3	84244	50.5	*P*>0.05
30–34	15793	26.8	44374	26.6	*P*>0.05
≥35	5716	9.7	15848	9.5	*P*>0.05
**Minority**					
Han	56754	96.3	160547	96.2	*P*>0.05
Others	2174	3.7	6272	3.8	*P*>0.05
**Smoking Status**					
Smoking	1503	2.6	4003	2.4	*P*>0.05
Second-hand Smoking	6417	10.9	16398	9.8	*P*>0.05
**Education level**					
Junior/Middle School	47779	81.1	136975	82.1	*P*>0.05
High School	10460	17.8	27759	16.6	*P*>0.05
College/University	689	1.2	2085	1.3	*P*>0.05

**Table 5:** Pregnancy Outcome among the Individuals who Participated/did not Participated in Preconception Health Check-up.

Pregnancy Outcome	Individuals who Participated in Check-up Service		Individuals who did not Participate in Check-up Service	
	Number	Percent (%)	Number	Percent (%)
Total Newborn	58928	97.6	166819	94.0
Normal Newborn	57779	98.1	165234	99.1
Male	30990	52.6	88138	52.8
Female	26789	45.5	77096	46.2
Prematurity	569	0.97	497	0.30
Low Birth Weight	557	0.95	954	0.57
Androgyneity	0	0.00	1	0.0006
Birth Defect *	23	0.04	134	0.08
Male	15	0.03	87	0.05
Female	8	0.01	46	0.03
Total Terminated Pregnancy #	1470	2.43	10597	5.97
Spontaneous Abortion	797	1.32	1282	0.72
Elective Abortion	350	0.58	6917	3.90
Abortion by Labor Induction	69	0.11	943	0.53
Ectopic Gestation	45	0.07	666	0.38
Fetal Death/Stillbirth	76	0.13	308	0.17
Others	133	0.22	481	0.27
In Total	60398	100.00	177416	100.00

*χ2 = 10.685, *P* = 0.001; #χ2 =  1820.17, *P*<0.0001.

### Birth defects in different regions of Hubei Province

**Figure 1:**
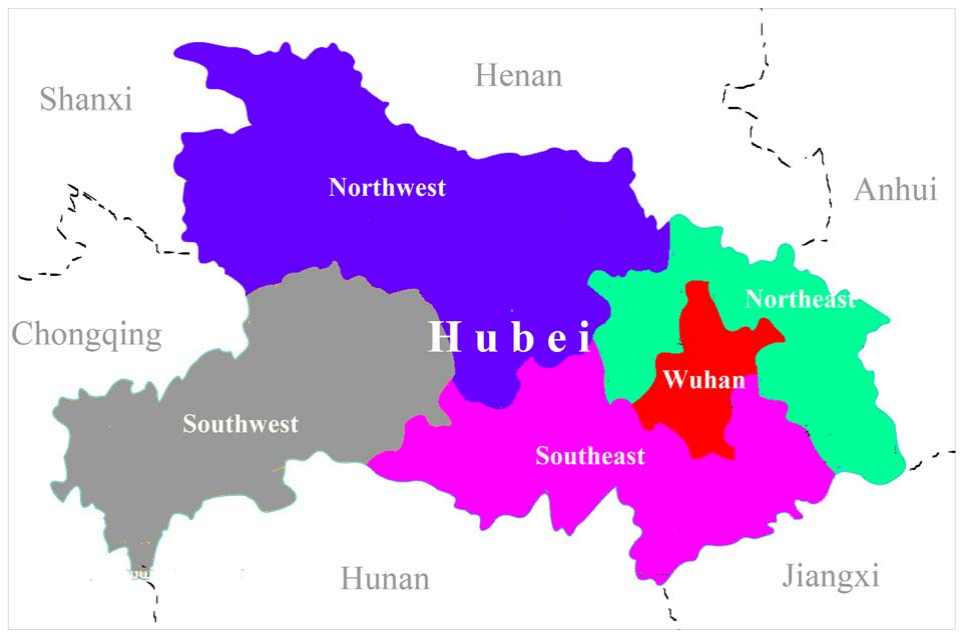
The map of Hubei Province, China. Wuhan, the capital city, and four regions of Hubei Province are highlighted with different colors.

The areas of Hubei Province are divided into five regions, mainly according to the location and economic development, which are Wuhan City (capital city of Hubei Province), Northeast, Northwest, Southeast and Southwest in Hubei Province (Fig. 1). The number and prevalence of birth defects in every region were summarized and compared respectively. The prevalence of birth defects among the parturient who participated in the service was lower than that among the parturient who did not participate in Northwest (χ2 = 6.77, *P* = 0.003) and Southwest (χ2 = 8.67, *P* = 0.009) (Table 6).

**Table 6:** Birth defect in different regions of Hubei Province.

Regions	Individuals who participated in the service				Individuals who did not participate in the service				
	Numbers of County	Number of Newborns	Birth Defect	Percent (%)	Number of County	Number of Newborns	Number of Birth Defect	Percent (%)	P value
Wuhan	7	6400	5	0.078	2	23848	39	0.160	*P* = 0.11
Northeast	9	13968	3	0.021	4	23155	6	0.026	*P* = 0.94
Southeast	11	18742	9	0.048	5	19090	27	0.140	*P* = 0.003
Northwest	9	7443	4	0.054	4	22399	43	0.190	*P* = 0.009
Southwest	10	12375	2	0.016	6	78327	19	0.024	*P* = 0.81
Total	46	58928	23	0.04	21	166819	134	0.08	*P* = 0.001

## Discussion

While prenatal care or maternal care and other interventions during pregnancy can address conditions that occur during pregnancy, they are not designed to address the high-risk factors to adverse pregnancy outcomes before pregnancy. Interventions to reduce the adverse pregnancy outcomes or improve the birth outcomes may need to start before pregnancy. Presently, preconception health care is strongly endorsed by the researchers and clinicians [17]–[19]. This study provides an overview of preconceptual screening examination service in rural areas of Hubei Province in 2012. The local family planning institutes provided the free preconceptual screening examination service for 497,860 individuals in rural areas, which covered 97.1% of the eligible individuals, higher than the coverage rates in 2010 (68.0%, 65,170/95,838) and 2011 (94.8%, 186,452/196,596). In the coming years, the government still need to invest more funding in this service to increase the coverage rate and cover the individuals not only in rural areas but also in the urban areas.

HPPFPC recently released 10 recommendations to improve preconception and primary health care for women and men. These recommendations included increased public awareness of the importance of preconception health behavior and preconception care services, as well as the provision of risk assessment and educational and health promotion counseling to all women of childbearing age to reduce reproductive risks and improve pregnancy outcomes in the event of pregnancy. An increasing number of couples were made aware of the importance of preconceptual screening examination and thus participated in this project. A total of 497,860 individuals participated in the free preconceptual screening examination service in 2012, but not all couples participated in the service together. Moreover, not all eligible individuals participated in this service. The couples living in remote areas might have less willingness for and interest in preconception health care. Thus, HPPFPC still needs to improve its service and strive to cover more couples, particularly in those remote areas. The local family planning institutes should put more effort into the propaganda of preconception health care, so that to raise the people's awareness of the importance of preconception health and people's self-consciousness to participate in this service.

After history taking, physical examination, and medical tests, the physicians would identify and assess the potential factors, including genetic, environmental, psychological, and behavioral factors, which might result in birth defects or other adverse pregnancy outcomes. For couples without risk factors, physicians would suggest that they come regularly to gain more health education and guidance. If only one partner participated in the service, physicians would suggest that the other partner participate as soon as possible. For those who had potential risk factors, physicians would inform the couple of the risk factors and potential effects on the fetus, aside from recommending further consultation, examination, referral, and treatment, while postponing childbearing if necessary. In 2012, 59,935 participants (12.04%) were grouped to the high-risk population and suggested to get further examination and treatment in hospital, which might help reduce the prevalence of adverse pregnancy outcomes. Also, preconceptual screening examination service might help to address the risk factors that could lead to adverse pregnancy outcome, so increased attention were given by the participants and physicians.

Although adequate prenatal, obstetric, and primary care services can reduce infant and maternal mortality, preconception health care refers not only to the primary prevention of maternal and perinatal morbidity and mortality, but also to a primary approach used to address various health issues [16], [20]. In this study, the number of pregnant and parturient who came for the pregnancy outcome follow-up increased in 2012 compared with that in 2010 (753 pregnant and parturient) and 2011 (24778 pregnant and parturient). However, there were some loss between the pregnant and parturient who came for the early pregnancy follow-up and pregnancy outcome follow-up, as some pregnant and parturient would prefer hospitals in the urban area for the delivery or abortion, instead of local family planning institutions. The prevalence of birth defect among the parturient who participated in pregnancy follow-up was lower than that among the parturient who did not, so was the prevalence of other adverse pregnancy outcomes. In details, the prevalence of birth defects in Northwest and Southwest in Hubei Province was lower among the parturient who participated in the service than that among the parturient who did not. However, determining whether preconceptual screening examination service can decrease the birth defect or other adverse pregnancy outcomes is difficult. Because the detailed data of every individual are not available now and the time of pregnancy follow-up is limited. Luckily, the project of preconceptual screening service have been still carried on among larger population in Hubei Province with some technological improvement. Also, analysis such as regression and correlation analysis will be done to determine the relationship between preconceptual screening examination service and prevalence of birth defect or adverse pregnancy outcomes in the future. Furthermore, the couples living in those remote areas and isolated from health care might have a higher prevalence. The number of spontaneous abortion might be under-reported, given that data were only obtained from local family planning institutions. Some pregnant with spontaneous abortion did not come for the early pregnancy follow-up, instead, they went to hospitals. The data about spontaneous abortion of the pregnant who did not participate in this service were only reported from the department of Obstetrics and Gynecology, not including the outpatient department, where an important proportion of the abortion were carried out.

As more women than before have access to education and information, are employed, have personal income and decision making power, and delay pregnancy, many opportunities are available to inform them about the need for preconception care, risk factors leading to birth defects, and the importance of a healthy reproductive life. A good opportunity for the health of the mother and the infant to improve if for any adverse condition to be identified and addressed before pregnancy [21]. A number of lifestyle modifications and medical interventions can be of benefit to maternal and neonatal health when applied prior to conception [8], [22]. These interventions include making a pregnancy plan, smoking cessation, supplementation with folic acid, cessation or moderation of alcohol intake, avoiding contact with toxic and hazardous substances, improvement of diabetic control, and maintaining healthy lifestyle and behavior [22]–[24]. Preconception health care is a good method for health promotion (including advice and education and screening tests) and risk assessment for every couple and should thus be implemented widely in China.

This study has a number of limitations. Firstly, not every individual participated in all the 19 screening tests for several reasons. For example, some participants might not have been aware that the service included 19 screening tests. Thus, the family planning institutes need to take some measures to ensure that participants complete the whole process. The 19 screening tests included in this service are the basic examinations that related to the pregnancy outcomes, and only RMB 240 ($ 39.45) are provided by national finance and local public finance. Tests, such as chromosome examination, Mediterranean anemia screening, and sex hormones, which are also important and known associations with adverse pregnancy outcomes, are not included in this service. Also, some other free medical service, such as HIV screening and premarital health care, are carried out in local Centers for Disease Control and Prevention (CDC) or local maternal and child health care hospital, not in the same institution. In the future, the funding invested by the government shall be increased to carry out more screening tests, and the cross-service and information exchange between different health care service institutions shall be coordinated and implemented. Secondly, this work only included physicians working in family planning institutes and does not represent the preconception care that may be provided by other primary care providers, such as obstetricians and gynecologists, midwives, and personnel working in maternal and child health-care institutes. Individuals who participated in the study may have had a higher interest in preconception health or greater concern about preconception care services, but this study could not represent all individuals. More studies in varying populations may validate the role of preconceptual screening examination service. This study did not assess the prevalence of adverse pregnancy outcomes among men who participated in the service, as we did not collect the data in this respect. Furthermore, the data generated by the “National Project of Free Preconceptual Screening Examination Service Information Management System” were not divided into male and female sections, such that the number of male and female participants in every item is unclear. Although it is important to assure the completeness of data, it is also important to report and show the data from the health management system to the readers when not all the data are publicly available. The results may has certain significance and provide a useful and meaningful suggestion to the government and concerned departments in the future.

Preconception health strategies include aspects related to awareness, knowledge, skills, motivation, opportunity, access, supportive environments, policy development, and ultimately, behavioral change [5], [25]. The provision of routine health promotion before conception may encourage changes to improve health and may be an opportunity to identify risk factors, such as infection, that can be treated before pregnancy begins [6]. However, preconception health care is not widely practiced in China despite being apparently acceptable to health professionals and to women of childbearing age. In the future, the expansion of access to preconception health risk assessment and counseling at community health centers and publicly funded primary care sites should been proposed as a strategy for improving preconception health and health care for women and men. This study presented the basic data about the status of preconceptual screening examination service in rural areas of Hubei Province in 2012, which provided the National Population and Family Planning Commission a valid scientific bases upon which to construct effective policies. More detailed data and analysis shall be studied in the future to better understanding the importance of preconception health care to pregnancy outcome.
